# COVID-19 Prevalence, Risk Perceptions, and Preventive Behavior in Asymptomatic Latino Population: A Cross-Sectional Study

**DOI:** 10.7759/cureus.10707

**Published:** 2020-09-29

**Authors:** Lina Karout, Ayna Serwat, Huda El Mais, Mohamad Kassab, Fatima Khalid, Brisandi Ruiz Mercedes

**Affiliations:** 1 Radiology, American University of Beirut Medical Center, Beirut, LBN; 2 Internal Medicine, Self Researcher, Collierville, USA; 3 Medicine, Liaquat National Medical College, Karachi, PAK; 4 Medicine, University of Ottawa Heart Institute, Ottawa, CAN; 5 Cardiology, Massachusetts General Hospital, Boston, USA; 6 Medicine, King Edward Medical University, Lahore, PAK; 7 Infectious Diseases, Self Investigator, Greenbelt, USA; 8 Infectious Diseases, Hospital Aristides Fiallo Cabral, La Romana, DOM

**Keywords:** asymptomatic, covid-19, risk perception, latinos

## Abstract

Aims

To determine the prevalence, level of coronavirus disease 2019 (COVID-19) risk perception attitude and preventive behavior implemented by the Latino population in the United States of America (USA).

Methods

This cross-sectional study was conducted between July 25 and August 25, 2020, and included asymptomatic Latino participants (n=410) with no current/previous COVID-19 within a religious community in Maryland. Participants answered a questionnaire consisting of three components: patient demographics/socioeconomic status, COVID-19 risk perception, and precautionary behavior. Additionally, a focused history taking and physical examination were performed, and nasal swabs for COVID-19 testing were obtained.

Results

Around 80% of our study population was 35 years and older, considerably healthy, with only about a third reporting history of chronic disease (~80%); most were females (~66%). Of our participants, 90% lived under poverty; only ~6% had made it to college. When asked about the likelihood of acquiring COVID-19, 97.3% stated they have a low risk of getting infected. However, as we asked about the risk of individuals living in their neighborhood, state, and country, the rates changed to moderate to high (78.4%, 86.3%, and 86.6%, respectively). When asked about preventive behavior, 71.2% stated they never wear masks and 85.4% mentioned they never keep social distance. Additionally, 76 (18.5%) tested positive for COVID-19, whereas 64 (84.2%) developed symptoms at follow-up, 57 (75%) were hospitalized, and 4 (5.2%) died.

Conclusions

Our study identified inadequate COVID-19 threat perception and lack of engagement in preventive behavior among a group of Latinos living in the USA. We believe that Latino communities across the USA are at markedly high risk of acquiring, spreading, and dying of COVID-19.

## Introduction

The severe acute respiratory syndrome coronavirus 2 (SARS-CoV-2) is the causative agent of the transmissible coronavirus disease (COVID-19), which was declared a pandemic by the World Health Organization (WHO) on March 11, 2020 [1]. As of September 28, 2020, 7,059,087 cases were reported in the United States of America (USA) [2]. The pandemic has resulted in the introduction of drastic changes and restrictions to prevent further spread of the virus such as social distancing, quarantine when necessary, mask-wearing, and deliberate hand hygiene [3]. Implementing these individual preventive behaviors requires a clear understanding of the actual risk of COVID-19 and the will to put in the effort and time to adapt to these changes [4]. This subject is of great importance for the Latino population, which is at increased risk of acquiring SARS-CoV-19 and having unfavorable outcomes [5].

Available data from the Centers for Disease Control and Prevention (CDC) has demonstrated that the highest rates of hospitalization and death from COVID-19 in the USA are among racial and ethnic minority groups when compared to non-Hispanic white groups [5]. This high risk is increased explicitly among non-Hispanic Black people, Hispanics, and Latinos, and American Indians/Alaska Natives [5]. In a CDC analysis of the data from 5,187,853 cases, race/ethnicity was available for 2,669,792 (51%) cases. From those, 29.2% (779,596) were Hispanic/Latinos, whereas 18.4% (490,378) were non-Hispanic/Black. This was further supported by a recent study in the Baltimore-Washington DC area that showed that Latinos had a significantly higher prevalence of COVID-19 compared with other ethnic groups [6]. The difference in the prevalence of COVID-19 among Latinos and other ethnic groups can be attributed to many given factors such as low socioeconomic status, overcrowded living conditions, inequalities, and limited healthcare access [7-9]. The vast majority of Latino people work on the front-line job sectors such grocery stores, waste management, cleaning/sanitation services, and food delivery, making them constantly exposed to people or surfaces potentially infected with the virus [7]. In addition, Latino communities are more prone to be living in a multi-generational household with overcrowded conditions, making it challenging to take safety measures to safeguard senior family members or isolate those who are sick if space in the house is inadequate.

Studies have shown that an adequate perception of COVID-19 risk is associated with better engagement in recommended preventive behavior such as social distancing [3,10]. On the contrary, populations with inadequate COVID-19 risk perception are less likely to commit to precautionary actions to avoid COVID-19 and are associated with higher infection rates, which have deleterious effects on the individual’s health, community health, and global value by worsening the pandemic [3,11]. This is of great importance, especially in vulnerable populations and high-risk ethnic groups such as the Latinos, where a higher prevalence of the infection will be associated with higher complications and mortality. To date, there is limited knowledge of the level of COVID-19 risk perception faced by Latinos in the USA and their level of engagement in preventive measurements recommended by the CDC to avoid SARS-CoV-2 infection. This could be a significant factor influencing the high prevalence of SARS-CoV-2 infection is this population. Therefore, our study aims to assess the prevalence of COVID-19 and evaluate COVID-19 risk perception and preventive behavior implementation in an asymptomatic Latino population in the USA. This will provide insight into the gaps in knowledge faced by Latinos and efforts needed to be taken to provide adequate education and social support to decrease the virus spread, morbidity, and mortality.

## Materials and methods

Population

The study population consisted of 410 Latino individuals recruited within a religious community in Maryland, USA, between July 25 and August 25, 2020. Patients aged 18 years or older with no known current or previous history of COVID-19 infection were included in the study. This study was approved by the Institutional Review Board, and informed consent was obtained.

Questionnaire

Participants who were found to be eligible to participate in the study sat for a semi-structured questionnaire/interview. Participants were briefed about the study's objective, risks, benefits, and voluntariness, after which written informed consent was signed. Each participant's interview took approximately 20 minutes and was conducted in Spanish or English, according to the participant’s preference. The questionnaire was implemented to assess four main components: patients’ demographics and socioeconomic status, COVID-19 risk perception, precautionary behavior, and presence of COVID-19 symptoms.

Patients’ demographics and socioeconomic status questions assessed variables such as age, sex, chronic diseases, marital status, educational level, household size, and annual income (quantified as lower or higher than the 125% federal poverty guidelines - the measure of income used by the U.S. government to determine who is eligible for subsidies, programs, and benefits) [12]. The COVID-19 risk perception component measuring the subjective understanding of COVID-19 threat perceived by each individual was assessed using a scale of 1 to 3, where 1 indicated low risk, 2 indicated moderate risk, and 3 indicated high risk. The precautionary behavior evaluating patients’ behavioral aspects during the pandemic was measured using a scale of 1 to 3, with 1 indicating never, 2 indicating sometimes, and 3 indicating always.

Physical examination and COVID-19 testing

We conducted a focused history and physical examination to evaluate the asymptomatic status of patients. Patients’ blood pressure, heart rate respiratory rate, pulse oximetry (SpO_2_), and the presence or absence of pulmonary auscultatory findings were evaluated. Additionally, patients were referred for COVID-19 polymerase chain reaction (PCR) testing in a nearby testing center.

Follow-up

After receiving COVID-19 test results, positive patients were contacted to be informed of their results and oriented to healthcare's next steps. Patients were referred to their primary care physician or the local health department for follow-up. Subsequently, we assessed for the development of related symptoms.

Statistical analysis

Analyses were conducted using SPSS Version 24 for Windows (IBM Corp., Armonk, NY, USA). All data measurements were compared employing an independent t-test for numerical variables and Pearson's correlation for categorical variables. Categorical variables are presented as frequencies with percentages, and continuous variables are presented as means ± standard deviations. Results were considered statistically significant if p-value was ≤0.05.

## Results

Of the 410 participants included in our study, 47.6% of the participants were aged 35-44 years followed by 45-54 years (19.3%) and ≥55 years (12.9%). Females accounted for 65.9% of the population, and 32.9% of total participants had a history of chronic disease, with hypertension (78.5%) and diabetes mellitus ([DM] 12.6%) being the most common. Almost 90% (89.3%) of participants were under 125% for poverty category. Also, 48.1% lived in a crowded house of six members. Of the participants, 93.2% stated to lose their job due to the COVID-19 pandemic. In addition, 74.1% had a close family member or friend infected with the virus (Table 1)

**Table 1:** Participants’ baseline characteristics and socioeconomic status. Count (%) COVID-19, coronavirus disease 2019; DM, diabetes; HTN, hypertension

		Total (n=410)	COVID-19 Negative (n=334)	COVID-19 Positive (n=76)	p-Value
Age, years	18-24	36 (8.8%)	9 (2.7%)	27 (35.5%)	0.001
	25-34	47 (11.5%)	40 (12.0%)	7 (9.2%)	
	35-44	195 (47.6%)	171 (51.2%)	24 (31.6%)	
	45-54	79 (19.3%)	68 (20.4%)	11 (14.5%)	
	≥55	53 (12.9%)	46 (13.8%)	7 (9.2%)	
Gender	Male	140 (32.1%)	125 (37.4%)	15 (19.7%)	0.003
	Female	270 (65.9%)	209 (62.6%)	61 (80.3%)	
Chronic disease	No	275 (67.1%)	221 (66.2%)	54 (71.1%)	0.413
	Yes	135 (32.9%)	113 (33.8%)	22 (28.9%)	
Type of chronic diseases	DM	17 (12.6%)	14 (12.4%)	3 (13.6%)	0.001
	HTN	106 (78.5%)	98 (86.7%)	8 (36.4%)	
	Asthma	7 (5.2%)	0 (0%)	7 (31.8%)	
	DM and HTN	5 (3.7%)	1 (0.9%)	4 (18.2%)	
Educational qualification	No education/illiterate	40 (9.8%)	36 (10.8%)	4 (5.3%)	0.001
	Primary school	256 (62.4%)	238 (71.3%)	18 (23.7%)	
	High school	88 (21.5%)	34 (10.2%)	54 (71.1%)	
	College	26 (6.3%)	26 (7.8%)	0 (0%)	
Relationship status	Single	51 (12.4%)	29 (8.7%)	22 (28.9%)	0.001
	Married/partner	286 (69.8%)	237 (71%)	49 (64.5%)	
	Divorced/separated	60 (14.6%)	57 (17.1%)	3 (3.9%)	
	Widowed	13 (3.2%)	11 (3.3%)	2 (2.6%)	
Economic status	<125% for poverty	366 (89.3%)	296 (88.6%)	70 (92.1%)	0.376
	≥125% for poverty	44 (10.7%)	38 (11.4%)	6 (7.9%)	
Household members	1-2	9 (2.2%)	6 (1.8%)	3 (3.9%)	0
	3-5	204 (49.8%)	184 (55.1%)	20 (26.3%)	
	6-8	121 (29.5%)	89 (26.6%)	32 (42.1%)	
	9-10	45 (11.0%)	31 (9.3%)	14 (18.4%)	
	≥11	31 (7.6%)	24 (7.2%)	7 (9.2%)	

When comparing COVID-19 positive and negative patients, we noticed that positive patients were significantly younger than negative patients (p<0.001). Interestingly, COVID-19 positive patients were more likely to be asthmatic (31.8% vs. 0% for negative), diabetic (13.6 vs. 12.4% for negative), and both diabetic and hypertensive (18.2% vs. 0.9% for negative) than negative patients (p<0.001). COVID-19 positive participants (69.7%) lived in overcrowded houses with six members significantly more than negative participants (43.1%) (p<0.001). Unpredictably, COVID-19 negative patients had a lower education level compared with positive patients, where 81.3% had no education or only accomplished primary school studies in negative patients versus 29% in positive patients (p<0.001).

 Table 2 represents the descriptive statistics of the risk perception of COVID-19 infection. Almost all participants (97.3%) believe that their risk of getting infected by COVID-19 is low, and 80% stated not to be worried at all. However, when asked about this risk to their neighbors, to the state, and the USA, 61% and 81% replied “moderate risk" and 75.6% replied “high risk", respectively.

**Table 2:** Descriptive statistics on the risk perception of COVID-19 infection. Count (%) COVID-19, coronavirus disease 2019

		Total (n=410)	COVID-19 Negative (n=334)	COVID-19 Positive (n=76)	p-Value
My probability of getting infected with COVID-19 is	Low	399 (97.3%)	331 (99.1%)	68 (89.5%)	0.001
	Moderate	10 (2.4%)	3 (0.9%)	7 (9.2%)	
	High	1 (0.2%)	0 (0%)	1 (1.3%)	
What level of threat do you think the COVID-19 represents for your work and finances?	Low	11 (2.7%)	9 (2.7%)	2 (2.6%)	0.389
	Moderate	17 (4.1%)	16 (4.8%)	1 (1.3%)	
	High	382 (93.2%)	309 (92.5%)	73 (96.1%)	
The risk of people from my neighborhood to get COVID-19 is	Low	89 (21.7%)	81 (24.3%)	8 (10.5%)	0.002
	Moderate	250 (61%)	190 (56.9%)	60 (78.9%)	
	High	71 (17.3%)	63 (18.9%)	8 (10.5%)	
The risk of people living in Maryland to get COVID-19 is	Low	56 (13.7%)	50 (15%)	6 (7.9%)	0.001
	Moderate	333 (81.2%)	273 (81.7%)	60 (78.9%)	
	High	21 (5.1%)	11 (3.3%)	10 (13.2%)	
The risk of people living in the USA to get COVID-19 is	Low	55 (13.4%)	47 (14.1%)	8 (10.5%)	0.001
	Moderate	45 (11%)	14 (4.2%)	31 (40.8%)	
	High	310 (75.6%)	273 (81.7%)	37 (48.7%)	
The risk of my co-workers to get COVID-19 is	Low	31 (7.6%)	27 (8.1%)	4 (5.3%)	0.001
	Moderate	74 (18%)	37 (11.1%)	37 (48.7%)	
	High	305 (74.4%)	270 (80.8%)	35 (46.1%)	
How worried are you about contracting COVID-19?	Low	328 (80%)	268 (80.2%)	60 (78.9%)	0.102
	Moderate	28 (6.8%)	19 (5.7%)	9 (11.8%)	
	High	54 (13.2%)	47 (14.1%)	7 (9.2%)	
How likely do you think you would meet someone who is infected with COVID-19?	Low	106 (25.9%)	94 (28.1%)	12 (15.8%)	0.026
	Moderate	0 (0%)	0 (0%)	0 (0%)	
	High	304 (74.1%)	240 (71.9%)	64 (84.2%)	
How worried are you that your family or friends might get infected with COVID-19?	Low	2 (0.5%)	0 (0%)	2 (2.6%)	0.001
	Moderate	343 (83.7%)	287 (85.9%)	56 (73.7%)	
	High	65 (15.9%)	47 (14.1%)	18 (23.7%)	

When comparing the different levels of COVID-19 risk perceptions of COVID-19 positive and negative patients, we noticed that negative patients (99.1%) thought their risk of having COVID-19 to be low significantly more than positive patients (89.5%) (p<0.001). Nevertheless, they had a better perception of the risk of COVID-19 at the level of their neighborhood and the USA compared with positive patients (p<0.05).

Table 3 represents the descriptive statistics of participants’ preventive behavior against COVID-19 infection. Almost 70% of participants answered “sometimes” when asked how often participants apply hand sanitizer and wash hands during the pandemic, with 2.9% saying never. Additionally, 71.2% mentioned never to wearing masks and 85.4% mentioned never to keeping social distance. Unfortunately, when asked if they would auto-isolate if necessary, 92.2% mention never.

**Table 3:** Descriptive statistics of COVID-19 preventive behavior. Count (%) COVID-19, coronavirus disease 2019

		Total (n=410)	COVID-19 Negative (n=334)	COVID-19 Positive (n=76)	p-Value
How often do you use hand sanitizer and wash your hands after COVID-19 started?	Never	37 (9%)	0 (0%)	37 (48.7%)	0.001
	Sometimes	287 (70%)	263 (78.7%)	24 (31.6%)	
	Always	86 (21%)	71 (21.3%)	15 (19.7%)	
How often do you avoid contact with high touch surfaces?	Never	12 (2.9%)	0 (0%)	12 (15.8%)	0.001
	Sometimes	325 (79.3%)	263 (78.7%)	62 (81.6%)	
	Always	73 (17.8%)	71 (21.3%)	2 (2.6%)	
How often do you use a mask?	Never	292 (71.2%)	264 (79%)	28 (36.8%)	0.001
	Sometimes	15 (3.7%)	4 (1.2%)	11 (14.5%)	
	Always	103 (25.1%)	66 (19.8%)	37 (48.7%)	
Would you agree to undergo temperature check-ups before entering public places if recommended?	Never	368 (89.8%)	306 (91.6%)	62 (81.6%)	0.001
	Sometimes	8 (2%)	1 (0.3%)	7 (9.2%)	
	Always	34 (8.3%)	27 (8.1%)	7 (9.2%)	
How often do you avoid going to crowded public places?	Never	59 (14.4%)	26 (7.8%)	33 (43.4%)	0.001
	Sometimes	322 (78.5%)	280 (83.8%)	42 (55.3%)	
	Always	29 (7.1%)	28 (8.4%)	1 (1.3%)	
If recommended, will you follow orders to self-isolate?	Never	378 (92.2%)	306 (91.6%)	72 (94.7%)	0.001
	Sometimes	4 (1%)	0 (0%)	4 (5.3%)	
	Always	28 (6.8%)	28 (8.4%)	0 (0%)	
How often do you avoid touching your face?	Never	48 (11.7%)	0 (0%)	48 (63.2%)	0.001
	Sometimes	327 (79.8%)	306 (91.6%)	21 (27.6%)	
	Always	35 (8.5%)	28 (8.4%)	7 (9.2%)	
How often do you keep social distance?	Never	350 (85.4%)	285 (85.3%)	65 (85.5%)	0.016
	Sometimes	31 (7.6%)	21 (6.3%)	10 (13.2%)	
	Always	29 (7.1%)	28 (8.4%)	1 (1.3%)	

When comparing the engagement in preventive behavior between COVID-19 positive and negative patients, we noticed that positive participants (never: 48.7%; sometimes: 31.6%; always: 19.7%) were significantly less likely to wash and use hand sanitizers compared with negative patients (never: 0%; sometimes: 79.7%; and always: 21.3%) (p<0.001). Similarly, positive patients (never: 15.8%; sometimes: 81.6%l and always: 2.6%) were less likely to avoid contact with touch surfaces compared to negative patients (never: 0%; sometimes: 78.7%; and always: 21.3%) (p<0.001). Intriguingly, positive patients (never: 36.8%; sometimes: 14.5%; and always: 48.7%) were significantly more likely to use face masks compared with negative patients (never: 79%; sometimes: 1.2%; and always: 19.8%) (p<0.001). When asked if they would self-isolate if recommended by health authorities, COVID-19 positive patients answered “never” (94.4% vs. 91.6% for negative) or “sometimes” (5.3% vs. 0% for negative) significantly more than negative patients (p<0.001). In addition, positive patients (never: 85.5%; sometimes: 13.2%; and always: 1.3%) were significantly less likely to keep social distancing compared with negative patients (never: 85.3%; sometimes: 6.3%; and always: 8.4%) (p<0.001).

Of 410 healthy, asymptomatic participants, 76 (18.5%) tested positive for COVID-19. All positive patients were contacted by phone to be informed about the results and for follow-up. Of 76 COVID-19 positive patients, 64 (84.2%) developed symptoms and 12 (15.7%) were still asymptomatic. Additionally, 57 (75%) had a more severe clinical picture requiring hospital admission. Of 57 hospitalized patients, 8 (14%) were admitted to the ICU and 49 (86%) to the wards. Of 76 positive COVID-19 patients, 4 (5.2%) passed away (Table 4).

**Table 4:** COVID-19 infected patients’ outcome Count (%) COVID-19, coronavirus disease 2019

		Total (n=76)
Follow-up of positive cases	Asymptomatic	12 (15.7%)
	Symptomatic	64 (84.2%)
Hospitalized/positive cases	No	19 (25%)
	Yes	57 (75%)
ICU admission	No	49 (86%)
	Yes	8 (14%)
Death	No	72 (95.7%)
	Yes	4 (5.2%)

## Discussion

COVID-19 is extensively being studied among minor ethnic communities in the USA and worldwide [13-15]. To our knowledge, this is the first study to determine the prevalence, level of risk perception, and preventative measures practice of COVID-19 in asymptomatic Latinos in Maryland and in the USA. Our study of 410 Latino adults compared the different baseline characteristics, level of risk perception, and preventive behavior between COVID-19 positive and negative patients. We established an inadequate level of risk perception in this minority group, which is significant in individuals testing positive for COVID-19 (p<0.001). Our data also demonstrated that a significant number of the COVID-19 positive participants had adopted only some preventive measures such as facemask wearing, with mostly abandoning other protective behaviors (i.e., hand washing and social distancing). These findings support that Latino minority groups are being disproportionately affected by COVID-19 in the setting of pre-existing unfavorable social determinants of health, such as poverty, low education, and healthcare access.

Around 80% of our study participants were 35 years and older and considerably healthy, with only about a third reporting history of chronic disease (~80% hypertension), the majority of which were females (~66%). Our data highlight a population of low socioeconomic and educational statuses, with 90% living in poverty, with only ~6% who having made it to college. Almost half of the participants of our participating community were living in densely populated households of six family members and more. When asked about their likelihood of acquiring an infection with COVID-19, nearly all individuals (97.3%) stated that they personally have a low chance of getting infected. However, as we slowly stretched the diameter to surrounding regions (i.e., neighborhood, state, and country) and asked about their impression of the risk on other individuals (i.e., neighbors, work partners, friends, and family), the majority of participants responded that others were at a moderate-to-high risk of getting infected with COVID-19. Also, 74% said they are very likely to come across someone infected. These findings are interesting and may translate to our population having a good understanding of the impact of the COVID-19 threat on the country and the general USA population; however, they somehow seemed to believe that they were more immune or at lower risk than other people. This could be due to optimism bias in which individuals tend to underestimate their risk of developing a disease and complications compared with other similar individuals [16].

A recent study performed in three different countries (the USA, United Kingdom, and Germany) suggested that optimism bias could affect the way people respond to the pandemic and the frequency of assumed preventive behaviors against COVID-19 [17]. In addition, our findings are consistent with a study conducted in the USA that compared COVID-19 awareness and concerns between different genders, races, and ethnicities. The study showed that women (27.9%), Black (36.15%), and Hispanic persons (32.8%), especially the ones living below the poverty level, were significantly more likely to state that they “were not at all likely” to get COVID-19 compared with males (19.7%), whites (17.5%), other races (29.45%), and non- Hispanics (22.3%) [18]. Another study contradicted our findings, where it showed high awareness of the possibility of being asymptomatically infected by SARS-Cov-2 in Hispanic and non-Hispanic Black individuals [19]. This difference could be explained by the fact that almost all of our population was under the same discrepancy with low socioeconomic and educational statuses. This low socioeconomic status is prevalent in the Latino population in the state of Maryland, and it is also very common that people with low socioeconomic status attend the religious groups looking for help and support. Gaps in the knowledge and awareness of COVID-19 in socioeconomically disadvantaged groups have been shown to affect the way individuals perceive and respond to the pandemic [18]. In the study by Jones et al., almost 85% of the population had at least some college education and an annual income of $30,000 or more [19]. Another possible explanation for this difference could be the fact that our population comes from a religious community that could result in giving a sense of protection toward COVID-19. In a late study, Qaiser et al. described how it may be challenging for healthcare professionals to achieve successful management of COVID-19 among different religious groups that would potentially replace preventative measures with their faithful beliefs, i.e., being protected by God and prayers would be enough for preventing and treating the infection [20]. In another study, spiritual leaders have been called to participate in the fight against COVID-19 to prioritize pandemic guidelines and assist in overcoming the stigma [21].

Participants were tested for COVID-19 infection, and, interestingly, 18.5% tested positive, 55.3% of which were 35 years and older, and almost 30% had a history of chronic disease (36.4% had hypertension and 31.8% had asthma). Minority ethnic groups are at higher risk of disease (particularly cardiovascular disease), and recent studies have shown that severe cases of COVID-19 infection in the elderly have been associated with multiple different underlying chronic health conditions including hypertension [22,23]. In addition to that, almost 70% of our positive COVID-19 cases were living in a crowded household. This may explain the high rates of COVID-19 positive patients in the group of participants living in overcrowded houses since it is challenging to maintain proper social distancing, face mask-wearing, and handwashing practices among family members [24]. These findings are alarming since these conditions make the situation difficult for COVID-19 positive participants to self-isolate and hence may explain our results where 94.7% of COVID-19 patients said that they will never be able to self-isolate if recommended, resulting in an additional risk on healthy household members.

There was a noticeable low perception of the risk of COVID-19 and inconsistent compliance with the government-recommended preventive measures in our study. This was noticed explicitly in COVID-19 positive patients. Although there was a high prevalence of participants who were consistent in wearing face masks, they stated they were less likely to wash/sanitize their hands and avoid contact/crowded places and comply with social distancing [25]. An interesting point here is that a significant number of COVID-19 positive participants were ones that reported always wearing their face masks (~50%). Based on these findings, our data may emphasize the importance of fully (vs. partially) adhering to the WHO- and CDC-recommended COVID-19 mitigation guidelines, including social distancing and proper hygiene [26]. Historically, a strong relationship has been witnessed between risk perception and precautionary behavior, which also applies to the COVID-19 pandemic, as shown in recent studies [17]. Individuals with better knowledge, awareness, and guidance on the risks and severity of consequences associated with the novel virus are more likely to adopt proper preventive measures [14]. Another interesting aspect of our study was the significantly higher incidence of positive cases in the female population when compared with males. It is common to have a higher female population among religious communities; however, it is uncommon to have higher positive cases when compared with the male population. This is a contrast with the findings in a recent study, where the COVID-19 cases were equally distributed among females and males, but the severe disease was most frequent among males. Additionally, when analyzing the population that required admission to ICU, all of them were males. It is demonstrated in the literature that males are more at risk of adverse outcomes regardless of age or comorbidities [27].

On follow-up of the 76 positive COVID-19 cases, we found that ~85%, ~75%, 14%, and ~5% were symptomatic, hospitalized, ICU admitted, and died. Is important to note that all the hospitalized and death patients were older and presented comorbidities such DM and asthma. These outcomes further warrant increased attention to the underprivileged Latino communities in the USA during the COVID-19 pandemic era. In general, nationwide Latino populations are at high risk for morbidities and mortalities due to the socioeconomic discrepancies and racial inequities that result in their lack of appropriate education, employment, housing, and inadequate access to healthcare and medical insurance [28]. Besides the evident socioeconomic impacts of COVID-19 and increased number of jobs lost, the persisting structural health disparities have shown increased numbers in COVID-19 diagnoses and deaths among the Latino populations and other ethnic minority groups across the USA (e.g., Black communities) [13]. This has been exacerbated by the multifactorial vulnerability of the underserved Latinos, putting them at a particular disadvantage during the COVID-19 pandemic.

Our study has some limitations. First, our data may contain selection bias since it focuses on a proportion of Latinos living significantly in poverty, which does not reflect the actual socioeconomic status of all Latinos across the USA. Another limiting factor is that all the collected data for our study were self-reported, which could have led to recall bias. Third, our sample was taken from a small-scale region and is based on observational and descriptive analyses. It would be useful to carry out a wide-ranging, heterogeneous data collection with more extensive analytical methods in the future.

## Conclusions

Our study identified concerning inadequate COVID-19 threat perception and lack of engagement in acceptable preventive behavior among a group of Latinos living in the USA. This provides a snapshot of the actual miscommunication and disadvantage present in this vulnerable group, which is under low socioeconomic status with limited educational levels. Therefore, we believe that Latino communities across the USA are at especially high risk of acquiring, spreading, and dying of COVID-19. We call for governments to come together with social service organizations and local media outlets to directly deliver COVID-19 information to their local populations in several non-English languages to overcome linguistic barriers. We argue that focused efforts should be put toward increased awareness, trusted educational resources, guideline implementation, financial and social support, and collaboration with faith-based organizations, and greater efforts should be taken to prioritize COVID-19 testing in at-risk populations. In our study, patients were asymptomatic, they did not have a clear knowledge about accessing healthcare in the case of COVID-19, and most of them were uninsured and were hesitant to access to healthcare due to lack of migratory status. This could contribute to the fact that most of them would be going late to receive medical care, resulting in higher mortality among the population. Due to all these factors, further research on COVID-19 among minority ethnic groups is needed.

## References

[1] Cucinotta D, Vanelli M (2020). WHO declares COVID-19 a pandemic. Acta Biomed.

[2] (2020). Coronavirus disease (COVID-19) pandemic. https://www.who.int/emergencies/diseases/novel-coronavirus-2019?gclid=EAIaIQobChMItJGS0c316wIViZWzCh328AUwEAAYASAAEgI-WvD_BwE.

[3] (2020). United States COVID-19 Cases and Deaths by State. Www.Cdc.Gov.

[4] Wise T, Zbozinek T, Michelini G, Hagan C, Mobbs D (2020). Changes in risk perception and protective behavior during the first week of the COVID-19 pandemic in the United States
[PREPRINT]. Psyarxiv Preprints.

[5] (2020). Update: Coronavirus case rates and death rates for Latinos in the United States. https://salud-america.org/coronavirus-case-rates-and-death-rates-for-latinos-in-the-united-states/.

[6] Martinez DA, Hinson JS, Klein E, Irvin N, Saheed M, Page K, Levin S (2020). SARS-CoV-2 positivity rate for Latinos in the Baltimore-Washington, DC region. JAMA.

[7] Calo WA, Murray A, Francis E, Bermudez M, Kraschnewski J (2020). Reaching the hispanic community about COVID-19 through existing chronic disease prevention programs. Prev Chronic Dis.

[8] Bibbins-Domingo K (2020). This time must be different: disparities during the COVID-19 pandemic. Ann Intern Med.

[9] Page KR, Venkataramani M, Beyrer C, Polk S (2020). Undocumented U.S. immigrants and COVID-19. N Engl J Med.

[10] Ferrer R, Klein WM (2015). Risk perceptions and health behavior. Curr Opin Psychol.

[11] Brown VJ (2014). Risk perception: it’s personal. Environ Health Perspect.

[12] (2020). HHS poverty guidelines for 2020. https://aspe.hhs.gov/poverty-guidelines.

[13] Pareek M, Bangash MN, Pareek N (2020). Ethnicity and COVID- 19: an urgent public health research priority. Lancet.

[14] Abuelgasim E, Saw LJ, Shirke M, Zeinah M, Harky A (2020). COVID- 19: unique public health issues facing Black, Asian and minority ethnic communities. Curr Probl Cardio.

[15] Millett G, Jones A, Benkeser D (2020). Assessing differential impacts of COVID-19 on black communities. Ann Epidemiol.

[16] Sharot T (2011). The optimism bias. Curr Biol.

[17] Kuper-Smith B, Doppelhofer Doppelhofer, Oganian Y, Rosenblau G, Korn C (2020). Optimistic beliefs about the personal impact of COVID-19 [PREPRINT]. Psyarxiv Preptrints.

[18] Wolf M, Serper M, Opsasnick L (2020). Awareness, attitudes, and actions related to COVID-19 among adults with chronic conditions at the onset of the U.S. outbreak: a cross-sectional survey. Ann Intern Med.

[19] Jones J, Patrick S, Travis S (2020). Similarities and differences in COVID-19 awareness, concern, and symptoms by race and ethnicity in the United States: cross-sectional survey. J Med Internet Res.

[20] Iqbal Q, Malik A, and Saleem F (2020). Religious cliché and COVID-19 management: a barrier for physicians. Br J Gen Pract.

[21] James A, Eagle L, Phillips C (2020). High COVID-19 attack rate among attendees at events at a church — Arkansas, March 2020. MMWR Morb Mortal Wkly Rep.

[22] Huang C, Wang Y, Li X (2020). Clinical features of patients infected with 2019 novel coronavirus in Wuhan, China. Lancet.

[23] Wu C, Chen X, Cai Y (2020). Risk factors associated with acute respiratory distress syndrome and death in patients with coronavirus disease 2019 pneumonia in Wuhan, China. JAMA Intern Med.

[24] Wang Y, Tian H, Zhang L (2020). Reduction of secondary transmission of SARS-CoV-2 in households by face mask use, disinfection and social distancing: a cohort study in Beijing, China. BMJ Glob Health.

[25] VoPham T, Weaver M, Hart J, Ton M, White E, Newcomb P. (2020). Effect of social distancing on COVID-19 incidence and mortality in the US [PREPRINT]. MedRxiv.

[26] Czeisler M, Tynan MA, Howard M (2020). Public attitudes, behaviors, and beliefs related to COVID-19, stay-at-home orders, nonessential business closures, and public health guidance — United States, New York City, and Los Angeles, May 5-12, 2020. MMWR Morb Mortal Wkly Rep.

[27] Jin j, Bai P, He W (2020). Gender differences in patients with COVID- 19: focus on severity and mortality. Front Public Health.

[28] Brewer NT, Chapman GB, Gibbons FX, Gerrard M, McCaul KD, Weinstein ND (2020). Meta-analysis of the relationship between risk perception and health behavior: the example of vaccination. Health Psychol.

